# Hypercapnia-Driven Skeletal Muscle Dysfunction in an Animal Model of Pulmonary Emphysema Suggests a Complex Phenotype

**DOI:** 10.3389/fphys.2020.600290

**Published:** 2020-10-29

**Authors:** Joseph Balnis, Chun Geun Lee, Jack A. Elias, Ariel Jaitovich

**Affiliations:** ^1^Division of Pulmonary and Critical Care Medicine, Albany Medical College, Albany, NY, United States; ^2^Department of Molecular and Cellular Physiology, Albany Medical College, Albany, NY, United States; ^3^Department of Molecular Microbiology and Immunology, Brown University, Providence, RI, United States

**Keywords:** pulmonary emphysema, COPD, hypercapnia, muscle dysfunction, muscle atrophy

## Abstract

Patients with chronic pulmonary conditions such as chronic obstructive pulmonary disease (COPD) often develop skeletal muscle dysfunction, which is strongly and independently associated with poor outcomes including higher mortality. Some of these patients also develop chronic CO_2_ retention, or hypercapnia, which is also associated with worse prognosis. While muscle dysfunction in these settings involve reduction of muscle mass and disrupted fibers’ metabolism leading to suboptimal muscle work, mechanistic research in the field has been limited by the lack of adequate animal models. Over the last years, we have established a rodent model of COPD-induced skeletal muscle dysfunction that allowed a disaggregated interrogation of the cellular and physiological effects driven by COPD from the ones unique to hypercapnia. We found that while COPD and hypercapnia synergistically contribute to muscle atrophy, they are antagonistic processes regarding fibers respiratory capacity. We propose that AMP-activated protein kinase (AMPK) is a crucial regulator of CO_2_ signaling in hypercapnic muscles, which leads to both net protein catabolism and improved mitochondrial respiration to support a transition into a substrate-rich, fuel-efficient metabolic mode that allows muscle cells cope with the CO_2_ toxicity.

## Introduction

Dysfunction of non-ventilatory skeletal muscles, which encompasses reduction of muscle mass and force-generation capacity, is a relevant comorbidity in patients with chronic pulmonary diseases including chronic obstructive pulmonary disease (COPD) (Jaitovich and Barreiro, 2018; Jaitovich et al., 2019, 2020). In the COPD population, emphysema phenotype has a greater association with muscle dysfunction than bronchial phenotype (Vanfleteren et al., 2013). Muscle dysfunction is strongly associated with higher mortality and other poor outcomes in these patients (Marquis et al., 2002; Swallow et al., 2007; Shrikrishna et al., 2012; Burtin et al., 2016); these associations persist even after adjusting for the magnitude of pulmonary disease and other covariables suggesting that muscle dysfunction could be independently responsible for the worse prognosis (Swallow et al., 2007; Shrikrishna et al., 2012). Moreover, very few interventions can improve muscle status in COPD (Vogiatzis et al., 2011), and none has shown mortality benefits (Casaburi and ZuWallack, 2009).

It is generally accepted that maximal force-generation capacity predominantly depends on muscle mass, and submaximal force, or endurance, on fibers metabolic properties (Sala et al., 1999; Richardson et al., 2004; van den Borst et al., 2013; Jaitovich and Barreiro, 2018). Both fibers’ mass and metabolism are disrupted in COPD; indeed, inferential models indicate that fibers metabolic disturbances are associated with higher mortality in COPD, even after multivariable correction for the magnitude of muscle atrophy (Patel et al., 2014). It is not known whether atrophy is a consequence of metabolic dysfunction or vice versa, or if they can occur independently of each other. A major limitation of research conducted in the field has been the lack of adequate animal models of COPD-induced skeletal muscle dysfunction, which has precluded any disaggregated interrogation of these processes. Specifically, animal models of COPD have been primarily generated to investigate the pulmonary condition that characterizes this disease and not the associated comorbidities (Campbell, 2000). Thus, current genetic, pharmacologic and toxic models of pulmonary emphysema are typically not calibrated to interrogate the skeletal muscle phenotype demonstrated by COPD patients (Gross et al., 1965; D’Armiento et al., 1992; Degens et al., 2015).

Chronic CO_2_ retention, or hypercapnia, occurs in many COPD patients, particularly in advanced stages of pulmonary disease (Brat et al., 2018). While we have recently reported mechanisms regulating loss of muscle mass (atrophy) in hypercapnia (Jaitovich et al., 2015; Korponay et al., 2020), research has also been significantly limited by the lack of an adequate animal model of COPD-driven muscle dysfunction that reminisces the multiple aspects present in humans. Therefore, information available on hypercapnia is largely observational or inferred from the effects of CO_2_ on otherwise healthy animals; and thus, the complex biological mechanisms regulating CO_2_-retaning COPD-driven muscle dysfunction are not well-understood.

The mouse model of COPD-driven muscle dysfunction we have established develops hypoxia but demonstrates normal pCO_2_ levels (Balnis et al., 2020a,b). By chronically exposing that animal to a hypercapnic environment, we were able to separately investigate normocapnic and hypercapnic COPD; that research has facilitated observations about the complex interaction between CO_2_ elevation and COPD muscles, which are presented in this article. These observations support current investigations based on the postulation that COPD and hypercapnia lead to muscle dysfunction in a non-linear fashion: while both COPD and hypercapnia synergistically contribute to muscle atrophy, they are antagonistic processes regarding fibers respiratory capacity. Evidence supporting these statements and future research avenues are presented in the following sections.

## Effects of Elevated Co_2_ on Skeletal Muscle Integrity

Although research focused on the combined effects of hypoxia and hypercapnia on skeletal muscle has been conducted (Shen and Huang, 2019), insights on the specific effects of elevated CO_2_ on skeletal muscle turnover was contributed by seminal work by Sharabi et al. (2009) conducted in *Caenorhabditis elegans*, who identified substantial CO_2_-induced, time-dependent disruption of body muscle organization and slowed development; both associated with an extension of the worms life span. Using cellular and *in vivo* models, both in mice and humans, we have later observed compelling evidence indicating that chronic hypercapnia regulates muscle turnover via AMP-activated protein kinase (AMPK).

### Hypercapnia Causes Accelerated Protein Catabolism

In chronically hypercapnic mice, we observed a reduction of animals’ weight, individual soleus and extensor digitorum longus (EDL) muscles weight, and force-generation capacity scored by the grip strength test (Jaitovich et al., 2015; Balnis et al., 2020c). These processes are associated with an atrophic phenotype as reflected by reduced muscle fibers cross-sectional area (CSA) and leftward shift of the fibers size distribution, which indicates muscles repopulation with smaller fibers upon elevated CO_2_ exposure (Jaitovich et al., 2015). To gain further insight into the possible mechanisms regulating hypercapnia-induced muscle atrophy, we exposed cultured myotubes to *in vitro* hypercapnic conditions for up to 2 days. These myotubes were maintained in buffered media in order to control normal pH and elevated CO_2_ conditions, as originally established by Vadasz et al. (2008) and replicated by others (Vohwinkel et al., 2011). Hypercapnic myotubes demonstrated a time-dependent reduction of transversal maximal diameter without evidence of cell death or toxicity; the same phenotype was observed in cells exposed to dexamethasone, a well-known atrophy-inducing drug (Jaitovich et al., 2015). As muscle atrophy typically results from accelerated muscle protein catabolism, and given that the ubiquitin-proteasome pathway is a major intracellular protein degradation system (Glickman and Ciechanover, 2002), we interrogated the expression levels of muscle specific E3 ubiquitin ligases in hypercapnia, and found that muscle-specific RING finger protein-1 (MuRF1) (Bodine et al., 2001) was also induced in a time-dependent fashion both in cultured myotubes and animals exposed to elevated CO_2_. Importantly, genetic silencing of *MuRF1* abrogated that fiber size reduction *in vitro*, and *MuRF1^–/–^* mice were found to be resistant to the muscle-catabolic effects induced by hypercapnia (Jaitovich et al., 2015). To investigate the mechanisms regulating that process, we focused on the possible role of AMPK, which had been previously shown to be robustly regulated by elevated CO_2_ in alveolar pulmonary cells (Vadasz et al., 2008). AMPK was an important candidate as it had been found to phosphorylate and thus regulate the activation of the transcription factor FoxO3a (Greer et al., 2007), which is a canonical inducer of MuRF1 (Sandri et al., 2006). Indeed, genetic silencing of both *AMPK* and *FoxO3a* prevented the CO_2_-induced myotubes diameter reduction and MuRF1 induction, and overexpression of *FoxO3a* constructs holding serine-to-alanine mutations of the AMPK-specific targeted sites led to the same effects, strongly suggesting that chronic CO_2_ elevation contributes to muscle atrophy via AMPK/FoxO3/MuRF1 (Jaitovich et al., 2015).

### Hypercapnia Causes Attenuated Protein Anabolism

CO_2_-induced AMPK-mediated accelerated muscle catabolism is teleologically consistent with the fact that AMPK represents a cellular stress sensor and thus, protein catabolism activated by AMPK contributes proteolysis-derived substrate to support other relevant cellular processes in an energetically challenged environment (Hardie et al., 2012). That rationale led to the investigation of possible hypercapnia effects on the regulation of ATP-consuming protein anabolism. Our observations made in human quadriceps muscle biopsies from hypercapnic patients demonstrated a striking reduction of pre-ribosomal RNA (pre-rRNA) compared with muscles obtained from normocapnic individuals; pre-rRNA is a surrogate of ribosomal biogenesis (Korponay et al., 2020). Moreover, an unbiased proteomic analysis of mice EDL muscles indicated a CO_2_-induced downregulation of structural constituents of the ribosome and translational machinery proteins (Korponay et al., 2020). Data from mice chronically exposed to hypercapnia confirmed the reduction of pre-rRNA expression; moreover, incorporation of puromycin to skeletal muscle, which reflects active protein synthesis, was also downregulated in the context of chronic hypercapnia (Korponay et al., 2020). These processes were replicated by exposure of two independent lines of cultured myotubes to elevated CO_2_ conditions. While CO_2_ causes activation of AMPK (Jaitovich et al., 2015; Balnis et al., 2020c; Korponay et al., 2020), *AMPK* silencing led to the abrogation of CO_2_-driven attenuated anabolism suggesting that hypercapnia mediates protein synthesis via AMPK-driven reduction of ribosomal gene expression. As AMPK regulation by mammalian target of rapamycin (mTOR) pathway has been reported to modulate ribosomal biogenesis and protein synthesis (Nader et al., 2005; Laplante and Sabatini, 2009), we investigated the potential role of that signaling pathway in the context of high CO_2_ exposure. Myotubes treated with rapamycin demonstrated a robust dephosphorylation of mTOR, yet no difference in mTOR phosphorylation was observed in the context of CO_2_ stimulation (Korponay et al., 2020). Moreover, while high CO_2_ causes robust and significant downregulation of pre-rRNA expression and puromycin incorporation, rapamycin exerts no significant effect in pre-RNA levels, yet it causes a significant reduction of puromycin incorporation (Korponay et al., 2020). These data suggest that CO_2_ leads to AMPK activation which negatively regulates ribosomal rRNA expression and protein synthesis, effects that are not mimicked by rapamycin-induced deactivation of the mTOR pathway. While AMPK was previously reported to regulate ribosomal gene expression via phosphorylation of the transcription factor TIF-1A (Hoppe et al., 2009; Cao et al., 2017), we found no evidence supporting this mechanism in CO_2_-induced anabolic attenuation. Indeed, we confirmed the expression of the TIF-1A product with Crispr/Cas9-directed gene flagging, which silencing did not attenuate downregulation of rRNA or puromycin incorporation in hypercapnic myotubes. Moreover, while previous research had also shown that AMPK regulates ribosomal biogenesis via KDM2A-mediated H3K36me2 demethylation in the rRNA gene, we entertained that this process could also be relevant in hypercapnia. However, siRNA-mediated KDM2A silencing failed to protect hypercapnia-exposed cells from depressed protein synthesis (Korponay et al., 2020).

## A Novel Window to Appreciate the Interaction of COPD and Co_2_ Elevation in the Setting of Skeletal Muscle Dysfunction

Data generated with experimental models based on CO_2_ exposure to otherwise healthy animals provided important mechanistic insight on the regulation of muscle turnover in that setting (Jaitovich et al., 2015; Balnis et al., 2020c; Korponay et al., 2020). However, clinical hypercapnia does not occur in isolation but in the context of an underlying pulmonary disease causing CO_2_ retention (Weinberger et al., 1989). Thus, the lack of hypercapnia research generated on a validated animal model of COPD that demonstrates substantial features of muscle dysfunction represents a major limitation that complicates capturing complex cellular and molecular processes occurring in that context.

### Animal Model of COPD-Induced Skeletal Muscle Dysfunction

As pulmonary emphysema phenotype has a greater association with muscle dysfunction than bronchial phenotype (Vanfleteren et al., 2013), mechanistic research focused on COPD-driven locomotor muscle dysfunction should be based on an animal model demonstrating histological evidence of pulmonary emphysema and airways obstruction physiology. Moreover, that animal needs to be inducible in order to minimize temporal confounders such as muscle development and age-related sarcopenia. The pulmonary phenotype needs to be robust in order to mimic the disease severity shown by the majority of COPD patients with muscle dysfunction (Kwan et al., 2019). The phenotype should also follow a specific trajectory in which muscle dysfunction occurs after, and not simultaneously with, the occurrence of pulmonary disease, to reflect a secondary COPD comorbidity. Importantly, muscle dysfunction should be multidimensional including morphologic, metabolic and functional disruptions appreciated in the clinical setting (Campbell, 2000; Jaitovich and Barreiro, 2018). We have recently reported such a model based on interleukin-13 (IL13) overexpression in Club cells, leading to inducible pulmonary emphysema (Zheng et al., 2000; Balnis et al., 2020a,b). This model deliberately does not involve cigarette smoking as this exposure leads to minimal weight and muscle loss (Toledo et al., 2012), causes muscle toxicity independently of pulmonary disease (Basic et al., 2012; Degens et al., 2015; Chan et al., 2019), and represents a single stimulus to an otherwise healthy animal. Moreover, it is widely accepted that smoking is not the main mechanism leading muscle dysfunction in COPD because clinical observations made on these patients and control subjects occur after matching them for smoking history (Maltais et al., 2014). As we discuss in the following sections, the exposure of this established animal model to hypercapnia has tapped into potential processes not necessarily observed with reductionist settings, which could lead to the identification of relevant cellular targets to antagonize muscle dysfunction.

### The Interaction Between COPD and CO_2_ Retention Has Uncovered a Complex Phenotype

**FIGURE 1:**
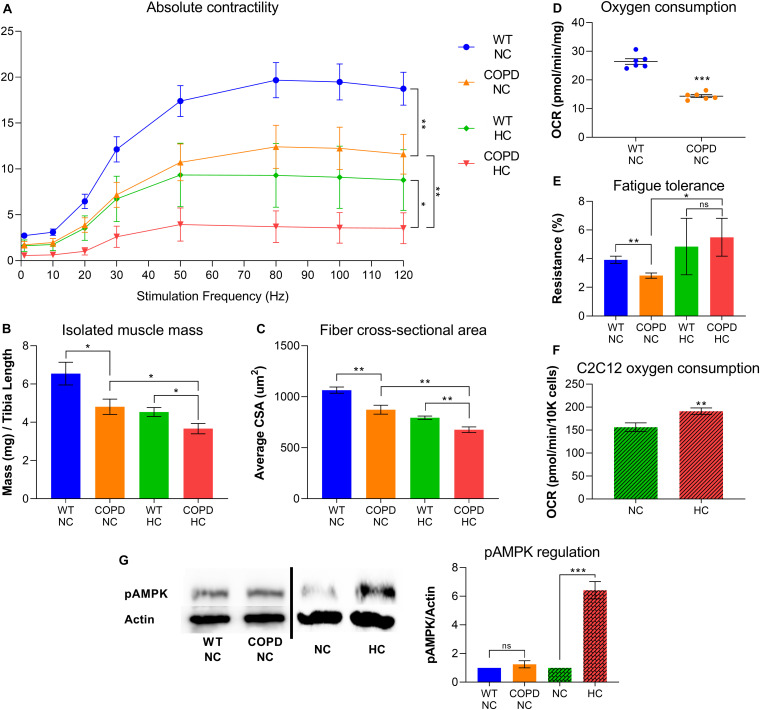
COPD and hypercapnia synergistically contribute to muscle atrophy, but they are antagonistic processes regarding fibers respiratory capacity. **(A)** Isolated EDL muscle contractility indicates that murine COPD (orange line) causes reduced absolute force-generation compared with wild type (WT) animal (blue line), which is incremented by hypercapnia (green line) and even further by the combination of hypercapnia and murine COPD (red line). Absolute force-generation capacity reflects muscle work dependent on muscle mass (*n* = 4). **(B)** Isolated EDL muscle mass and **(C)** fibers average cross-sectional area (CSA) follow the same incremental pattern of COPD and hypercapnia described in **(A)** (*n* = 4). **(D)** Oxygen consumption rate (OCR) by plate respirometry obtained with Seahorse^§^ technology demonstrates that EDL muscle reduced respiratory capacity induced by normocapnic murine COPD (*n* = 6). **(E)** Fatigue-tolerance, which partially depends on the fibers oxidative capacity, is reduced in normocapnic (NC) murine COPD versus wild type (NC-WT) animal (*n* = 4). However, the exposure of these animals to hypercapnic (HC) conditions leads to an improvement of their fatigue-tolerance (*n* = 4). **(F)** OCR by plate respirometry obtained with Seahorse^§^ technology indicates that C2C12 cells grown for 48 h in hypercapnic conditions demonstrate elevated respiratory capacity (*n* = 7). **(G)** Activation of AMPK, as reflected by its phosphorylation at threonine 172 (pAMPK) is not evident in the COPD animal but robustly demonstrated by muscles from animals exposed to hypercapnia (*n* = 4), *p < 0.05, **p < 0.01, ***p < 0.001. For technical details about the methods, see our recent publications (Jaitovich et al., 2015; Balnis et al., 2020a,b,c; Korponay et al., 2020). Presented data is original and thus has not been previously reported.

Clinical evidence indicates that COPD leads to skeletal muscle atrophy (Barreiro and Jaitovich, 2018; Jaitovich and Barreiro, 2018), and our recently established animal model consistently recapitulates that feature (Balnis et al., 2020a,b). Similarly, our research demonstrates that elevated CO_2_ leads to net muscle loss (Jaitovich et al., 2015; Balnis et al., 2020c; Korponay et al., 2020). We have recently found that both COPD and CO_2_ retention synergistically contribute to reduced force-generation capacity and muscle atrophy (Figures 1A–C). However, the combined metabolic effects of COPD and CO_2_ retention are more complicated: while COPD leads to reduced oxygen consumption and fatigue-tolerance (Figures 1D,E), plate respirometry analysis by Seahorse^®^ technology indicates that cultured hypercapnic muscle cells demonstrate increased oxygen consumption rate (OCR) (Figure 1F). Moreover, the COPD animal model exposed to chronic hypercapnia shows improved fatigue-tolerance compared with COPD normocapnic model (Figure 1E); fatigue-tolerance is critically dependent on the muscle fiber oxidative potential (Jaitovich and Barreiro, 2018; Balnis et al., 2020a). We have previously shown that hypercapnic mice EDL muscles demonstrate higher abundance of type-I (oxidative) fibers compared with normocapnic counterparts (Balnis et al., 2020c), which further provides evidence of a CO_2_-induced metabolic reconfiguration in skeletal muscle. Interestingly, a similar finding of an increase of type-I fibers has been previously described in hypercapnic rats (Shiota et al., 2004). Thus, this data suggests that while COPD and hypercapnia *synergistically* contribute to muscle atrophy, they are *antagonistic* processes regarding fibers respiratory capacity.

## Discussion

The complex interaction of COPD and hypercapnia regarding muscle atrophy and fibers oxidative capacity suggests CO_2_ could selectively activate optimization of mitochondrial respiration in this setting. Indeed, a recent analysis of EDL muscles proteome from chronically hypercapnic mice shows that the bioenergetics-related “ATP binding” is the most significantly downregulated term compared to normocapnic controls (Korponay et al., 2020), suggesting CO_2_-induced energetic cellular stress. Previous evidence indicates that high CO_2_ exposure causes epithelial cells short-term mitochondrial dysfunction and reduced ATP-generation capacity (Vohwinkel et al., 2011; Kryvenko et al., 2020); however, hypercapnia effects on skeletal muscle have so far been focused on muscle size and not on cellular metabolism (Jaitovich et al., 2015; Korponay et al., 2020). Moreover, even though AMPK senses hypoxia in some models (Gusarova et al., 2011; Hardie et al., 2012), and our COPD animal model is hypoxic (Balnis et al., 2020a,b), we have found no AMPK activation reflected by AMPK phosphorylation at threonine 172 (pAMPK) in COPD mouse muscles (Figure 1G), which is consistent with COPD patients muscle biopsies data (Guo et al., 2013; Natanek et al., 2013). By contrast, AMPK is robustly activated in hypercapnia (Vadasz et al., 2008; Jaitovich et al., 2015; Balnis et al., 2020c; Korponay et al., 2020) (Figure 1G); which supports the concept that AMPK represents a specific mediator of CO_2_ muscle signaling in the subgroup of hypercapnic COPD patients. Importantly, oxidative capacity correlates with the expressed isoform of myosin heavy chain: type I-expressing fibers have a higher oxidative capacity and are more fatigue-resistant than type II fibers (Schiaffino and Reggiani, 2011). As mentioned before, we found that hypercapnia causes an increase in type I (oxidative) fibers (Balnis et al., 2020a), supporting the concept of CO_2_-induced oxidative optimization. Seminal research conducted in AMPK double knockout (β1β2-KO) mice established the relevance of AMPK in maintaining skeletal muscle oxidative metabolism (O’Neill et al., 2011). Moreover, AMPK has been shown to drive mitochondrial biogenesis via peroxisome proliferator-activated receptor gamma coactivator 1-α (PGC-1 α) which operates via translational (Jager et al., 2007) and post-translational mechanisms such as AMPK-driven phosphorylation (Jager et al., 2007) and deacetylation (Canto et al., 2009). As PGC-1 α axis controls the fiber’s transformation from type II to type I (Lin et al., 2002; Jager et al., 2007), we speculate that CO_2_ triggers AMPK- PGC-1 α activation in skeletal muscle, which leads to a global metabolic reconfiguration characterized by a more oxidative phenotype which facilitates fibers respiration operating under stress. This model implicates AMPK as a necessary regulator of both net protein catabolism and improved mitochondrial respiration to support a transition into a substrate-rich, fuel-efficient metabolic mode that allows muscle cells cope with CO_2_ toxicity.

*In vivo* loss of AMPK function has been complicated by the fact that isoform-specific AMPK knockout mice maintain substantial residual activity of the non-ablated isoform, which dampens the metabolic phenotype demonstrated by the model (Mu et al., 2001; Jorgensen et al., 2004; Fujii et al., 2005; Maarbjerg et al., 2009). Thus, mechanistic studies accounting for that redundancy are needed to define whether AMPK regulates CO_2_-induced metabolic optimization in skeletal muscle.

### Other Possible Mechanisms Regulating CO_2_-Induced Metabolic Reconfiguration

Autophagy regulates muscle turnover (Masiero et al., 2009), and clinical evidence indicates that it is dysregulated in locomotor skeletal muscles from COPD patients (Guo et al., 2013; Hussain and Sandri, 2013). Autophagy can be regulated by AMPK (Masiero et al., 2009; Guo et al., 2013; Hussain and Sandri, 2013). Although we have reported that CO_2_ does not regulate the canonical autophagy switch mTOR in skeletal muscle (Korponay et al., 2020), it is possible that it controls AMPK-autophagy axis via an alternative mechanism. For instance, AMPK-activated mitophagy (Egan et al., 2011), which is an essential quality-control measure that prevents reactive oxygen species (ROS) formation (Garcia-Prat et al., 2016), could be a potential mechanism leading to higher mitochondrial respiration in hypercapnia. Also, AMPK has been recently reported to modulate TET2-dependent DNA methylation (Wu et al., 2018) and histone deacetylation (Chen et al., 2015), which could also link hypercapnia-AMPK with muscle metabolic reconfiguration via selective expression of genes needed to support oxidative capacity. Prolyl-hydroxylases (PHDs) are 2-oxoglutarate-dependent dioxygenase (2-OGDD) enzymes critical in the regulation of the transcription factor Hypoxia-inducible factor 1 (HIF-1) signaling, a master regulator of O_2_ homeostasis (Prabhakar and Semenza, 2012). The improved oxidative environment driven by elevated CO_2_ could alter the ratios of intermediate metabolites regulating PHDs such as succinate and fumarate (Weinberg et al., 2019; Martinez-Reyes and Chandel, 2020). This process, which can occur even in the normoxic environment (Selak et al., 2005), could interact with wasting signals by activating hypoxia-response elements (HREs) in the genome. Thus, the investigation of hypercapnia in the context of COPD-induced skeletal muscle dysfunction represents a unique opportunity to capture complex processes not accessible via highly reductionist settings.

### Muscle Fibers-Extracellular Matrix Coupling

**FIGURE 2:**
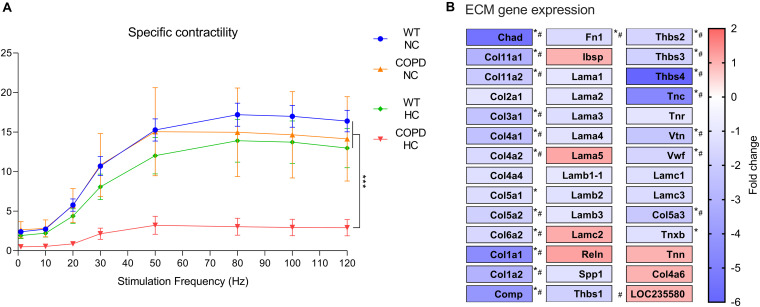
The combination of COPD and hypercapnia leads to a reduced specific force generation capacity. **(A)** While normo and hypercapnia *per se*, and normocapnic (NC) murine COPD associate with a preserved specific force-generation capacity, the combination of murine COPD and hypercapnia (HC) leads to a significant reduction of specific force, which is a surrogate of intrinsic contractile properties of individual fibers (*n* = 4), ***p < 0.001. **(B)** RNA seq analysis of EDL muscles obtained from normo and hypercapnic murine COPD model demonstrate a significant hypercapnia-induced downregulation of extracellular matrix (ECM) receptor interactions term and multiple genes. Sequencing was generated by an Ion Torrent Ion S5 plus system, Thermo Fisher scientific (*n* = 4), *p < 0.05; ^#^*p* > 1.5-fold change. For details about the animals details including genetic backgrounds and hypercapnia setup, see our recent publications (Jaitovich et al., 2015; Balnis et al., 2020a,b,c; Korponay et al., 2020). Presented data is original and thus has not been previously reported.

Our previous data supports the concept that muscle weakness in COPD is associated with a decreased absolute but not specific contractility (Balnis et al., 2020a,b), suggesting that reduced muscle force-generation capacity is due to atrophy and not an intrinsic contractile deficit of individual muscle fibers (Park et al., 2012). However, very recent data from our laboratory indicates that the combination of COPD and hypercapnia leads to a striking and non-incremental decrease in specific contractility (Figure 2A), suggesting inability of individual fibers to mount adequate contraction which is independent of their atrophy magnitude. While we do not observe any conspicuous histological alteration of muscle fibers integrity in the combined COPD/CO_2_ setting, a recent unbiased analysis of RNA sequencing data obtained from these animals’ EDL muscles shows a downregulation of various genes related to extracellular matrix (ECM) receptor interaction (Figure 2B). Interestingly, consistent previous evidence demonstrates that intramuscular connective tissue (IMCT) connections with the ECT facilitates lateral transfer of muscle force and contributes a substantial fraction of the muscle fiber force-generation capacity (Huijing et al., 1998). We speculate that hypercapnia-induced disruption of fiber-ECM interactions could undermine the coupling of fibers’ contraction with surrounding tissues leading to a reduction in specific force-generation capacity (Csapo et al., 2020). Future mechanistic research could define whether this finding is relevant to explain the observed phenotype.

## Conclusion

COPD and hypercapnia are frequently associated entities, and both contribute to skeletal muscle dysfunction. However, their interaction has so far not been mechanistically explored. By using an animal model of COPD-induced muscle dysfunction, we made observations that support the concept that COPD and hypercapnia synergize regarding muscle atrophy but antagonize on their respiratory effects. We hypothesize that AMPK is a critical mediator of this process, which supports a cellular environment operating under metabolic stress. Future research with AMPK loss-of-function analyses can elucidate the implications of these observations, which could be consequential to improve the management of hypercapnic COPD patients.

## Data Availability Statement

The raw data supporting the conclusions of this article will be made available by the authors, without undue reservation.

## Ethics Statement

The animal study was reviewed and approved by Albany Medical College Institutional Animal Care and Use Committee.

## Author Contributions

JB conducted the experiments leading to the presented figures. AJ wrote the manuscript. All authors edited the manuscript.

## Conflict of Interest

The authors declare that the research was conducted in the absence of any commercial or financial relationships that could be construed as a potential conflict of interest.
